# Beyond Right and Wrong: Fostering Connection in Emotion Theory Debates

**DOI:** 10.1177/17456916251319042

**Published:** 2025-03-11

**Authors:** Karlijn van Heijst, Annemie Ploeger, Mariska E. Kret

**Affiliations:** 1 Cognitive Psychology Unit, Faculty of Social and Behavioral Sciences, Leiden University; 2 Comparative Psychology & Affective Neuroscience Lab, Cognitive Psychology Unit, Leiden University; 3 Department of Psychology, University of Amsterdam; 4 Leiden Institute for Brain and Cognition, Leiden University

**Keywords:** emotion, affect, comparative psychology

## Abstract

Basic emotion theories (BETs) and the theory of constructed emotion (TCE) have both made significant contributions to the field of affective science despite a persistent divide between the two camps. We argue that focusing on which camp is right hampers possibly fruitful collaborations between affective researchers working within different theoretical frameworks. The TCE and BETs can complement each other because they focus on different features of and questions about affective processes. Clearly defining and operationalizing these questions is crucial to further elucidating the evolutionary basis of emotion and feeling.

Basic emotion theories (BETs) and the theory of constructed emotion (TCE) are two distinct frameworks that have independently made significant contributions to the field of emotion research. However, the persistent divide between the two camps has hindered progress rather than fostering it. This divide creates an unwelcoming environment for the next generation of researchers who aspire to engage in open discussions, collaborate, and seek common solutions. Although disagreements are natural and even essential for scientific advancement, it is crucial that such debates prioritize the pursuit of knowledge and the advancement of science over anything else. Only through constructive dialogue and mutual respect can the field truly move forward.

This has been, and continues to be, our goal. We therefore purposefully steer clear of engaging in a debate between who is right and who is wrong because such an approach keeps researchers “in their own lane” and does nothing to advance the field of affective science. As researchers who are not strictly aligned with either the BET or TCE framework—recognizing the merits of both—we are dedicated to finding common ground between these two perspectives despite their foundational differences. After having conducted our literature review (van Heijst et al., 2023), we still firmly believe that taking an evolutionary perspective on BETs and the TCE provides a promising avenue for bridging the divide between them. By fostering open dialogue and identifying shared principles, we aim to advance a more unified and collaborative approach to emotion research.

In their commentary on our article, Barrett et al. (2025) discussed what they perceive as certain “errors” in our work. Here, we provide a response, highlighting the important nuances we have presented and emphasizing the value of considering diverse viewpoints.

## Emotion and Feeling

Much of the disagreement in affective research stems from the imprecise and inconsistent usage of terminology—a point that others have also highlighted. Additionally, psychologists often conceptualize affective processes specifically within the context of human experience, whereas researchers studying affective processes in other species may adopt broader or differing perspectives.

To address this foundational issue, we begin by clarifying two critical terms that often dominate these discussions, “emotion” and “feeling,” and the umbrella term “affective process”: *Emotion* is the bioregulatory response to an external event that promotes survival (Damasio, 2004), *feeling* is the mental representation of the physiological changes that occur during an emotion (Damasio, 2004), and an *affective process* comprises features of both. Defined in this way, feeling is intrinsically linked to emotion, as argued by Barrett et al. (2025).

However, emotion is not necessarily dependent on feeling. In healthy human adults, we have the capacity to study both emotion and feeling, allowing us to explore how the two are interconnected. Although studying feeling in humans poses challenges—such as the unreliability of self-reported data—investigating feeling in nonhuman animals is even more difficult. Yet with emerging techniques, including advancements in artificial intelligence, we may be on the verge of overcoming these barriers and achieving a deeper understanding (see Note on Evolution section).

One of the key findings of our literature review is that BETs and the TCE tend to focus on different aspects of affective processes. Whereas BETs primarily emphasize emotion, the TCE places greater emphasis on feeling (van Heijst et al., 2023). However, this distinction is often obscured in the broader literature, in which these terms are frequently used interchangeably. Clearly delineating which aspect of affective processes is under study or discussion will be essential for building bridges between these theoretical frameworks and advancing the field of affective science. This clarity can help mitigate misunderstandings and foster more productive collaboration.

## Connecting the Theory of Constructed Emotion and Basic Emotion Theories

Tinbergen’s four “why” questions provide a comprehensive framework for understanding behavior by addressing its current function (adaptive purpose), evolution (phylogenetic history), causation (mechanistic processes), and development (ontogeny over an organism’s life span; Bateson & Laland, 2013; Tinbergen, 1963/2005). Comparing the TCE and BETs using these questions allows for a detailed analysis of what types of questions both theories ask about affective processes and therefore what is at the root of the disagreement between them. We conclude that BETs and the TCE not only tend to focus on different features of affective processes, emotion and feeling, but also on different questions about these features: BETs focus on the evolution question of emotion, and the TCE focuses on the causation of feeling. Specifically in regard to their evolutionary basis, our comparison further shows why it is difficult to integrate them at this level: We propose the evolution question of emotion is different from the evolution question of feeling (van Heijst et al., 2023). However, the theories do provide strikingly similar answers to the survival value/function question of affective processes: They guide appropriate action to increase survival and the well-being of the individual. Although the detailed answers might be different (see Barrett et al., 2025, Table 1), we underline that the general proposed function is the same. We do not mean to imply that this function is the main question to be asked about affective processes. However, it does suggest that the BET and TCE approaches to affective science could be complementary, especially considering their focus on different features of affective processes (van Heijst et al., 2023).

Considering what both theories can add to affective science, an important contribution of the TCE is a focus on the variability of concepts and categories used to describe feeling and, accordingly, the variation in bodily processes and context that accompanies them (e.g., Barrett, 2009; Barrett et al., 2025; Lindquist et al., 2012; Siegel et al., 2018). Moving beyond a strict adherence to basic emotion categories, we propose that BETs’ focus on the mechanistic aspects of emotion can provide a different angle. To better understand affective processes as a whole, we can, for example, study aspects of emotion such as pupil dilation, hand gestures, and facial movements across contexts and species. This can provide insights into a possible function of these mechanistic aspects of their own, as well as serving varying functions in affective processes as a whole. For example, the mechanistic action of retracting the lips and corners of the mouth typical of the human smile appears to serve a strikingly similar submissive/appeasement function in aggressive contexts across primate species (e.g., Bout & Thierry, 2005; Kim et al., 2022; Vlaeyen et al., 2022; Waller & Dunbar, 2005), including humans (Martin et al., 2017; Rychlowska et al., 2017). So although producing a smile might be variably associated with many different feelings, certainly not only with the concept of being “happy,” in specific contexts it seems to be associated with a particular function.

## Note on Evolution

When it comes to the evolution question, the divide between BETs and the TCE can be linked to the divide between the modern synthesis and the extended evolutionary synthesis in evolutionary psychology (Barrett et al., 2025; van Heijst et al., 2023). In our view, however, this comparison highlights the importance of finding common ground. In a highly cited article, Laland et al. (2015) stated that “the ‘extended evolutionary synthesis’ . . . retains the fundaments of evolutionary theory, but differs in its emphasis on the role of constructive processes in development and evolution, and reciprocal portrayals of causation” (p. 1). Laland et al. thus explicitly argued that we need to combine different approaches and shift the emphasis. The argument is not that the extended evolutionary synthesis abandons the modern synthesis or that the two are fundamentally different but that—just as the name implies—the modern synthesis should be extended. And just as Laland’s (2017) book is called *Darwin’s Unfinished Symphony*, Darwin’s “symphony” needs to embrace modern insights. We completely agree with that, and that is why we argue that both approaches are important.

To some extent, we agree with Barrett et al.’s (2025) caution against applying human emotional categories to behaviors in other species, such as chimpanzees. Chimpanzees are a distinct species with their own unique emotional repertoire that we may not fully recognize or understand because of the limitations of interpreting their behaviors through a human lens. When studying nonhuman animal emotions it is helpful to explicitly consider how inferences are being made from the study of human emotions (Mendl et al., 2022). However, we also argue for the importance of considering similarities between species and for using the same clearly defined and operationalized terms for behaviors that have similarities in form and function between human and nonhuman animals (Andrews, 2020; de Waal, 2019). We also need to consider similarities from our evolutionary past. Specifically comparing humans to chimpanzees, we can start by considering our shared evolutionary history until 6 to 8 million years ago (Langergraber et al., 2012). This implies that our affective processes evolved in the same way for billions of years, which likely leaves its traces in similarities between our species today. Further, for most of their evolutionary history, *Homo sapiens* lived as hunter-foragers until in some regions of the world the agricultural revolution radically transformed their ecological niche, starting around 12,000 years ago (Belfer-Cohen & Goring-Morris, 2011; Goring-Morris & Belfer-Cohen, 2011). This suggests that our brains evolved in an environment vastly different from the one we inhabit today yet strikingly similar to the natural habitats of wild chimpanzees. These evolutionary pressures have important implications for how our brains function today, most certainly influencing all aspects of affective processes.

Empirical evidence is still currently limited within both the TCE and BETs, especially when it comes to the evolutionary basis of affective processes. A comparative, multimethod approach used by researchers within different theoretical traditions can give us insights into the emotions and feelings of a variety of species. Further investigating affective processes in chimpanzees and bonobos, our closest living relatives, will be crucial. Although much remains unknown, new groundbreaking techniques provide promising new ways of noninvasively studying emotion and possibly feeling. Arousal levels, for example, can be measured with pupil dilation and thermography during natural behavior and/or while performing computerized tasks (for a review, see Nieuwburg et al., 2021). Further, cutting-edge screen-based experiments, such as eye tracking and touchscreen tasks, can possibly also tell us something about how the apes feel (for further discussion, see Kret, 2024; Kret et al., 2022). By working together toward gathering more data, we will be able to further piece together the evolutionary history of affective processes as a whole.

## Conclusion

We provided a nuanced comparison of BETs and the TCE in an attempt to underline that both approaches have their merit despite being fundamentally different. The TCE’s discussion of feeling underlines its complex interrelation with emotion and the importance of considering context and variation. BETs inspire a detailed investigation of the bioregulatory responses of emotion that, especially when studied comparatively, can lead to new functional insights. Striving to be open to the nuances in different views and curious about the types of evidence they can provide is ultimately the way forward.

## Transparency

*Action Editor:* Kent Berridge

*Editor:* Interim Editorial Panel

*Author Contributions*

K. van Heijst wrote the first draft, and A. Ploeger and M. E. Kret provided feedback on the manuscript. All authors approved the final manuscript for submission.
